# Knockdown of TPT1-AS1 inhibits cell proliferation, cell cycle G1/S transition, and epithelial–mesenchymal transition in gastric cancer

**DOI:** 10.17305/bjbms.2020.4470

**Published:** 2021-02

**Authors:** Jun Tang, Fei Huang, Hui Wang, Feng Cheng, Yaping Pi, Juanjuan Zhao, Zhihong Li

**Affiliations:** 1 Department of General Surgery, The Center Hospital of Ezhou, Ezhou, China; 2 Department of Medical Laboratory, The Center Hospital of Ezhou, Ezhou, China; 3 Department of Medical Laboratory, The Central Hospital of Wuhan, Tongji Medical College, Huazhong University of Science and Technology, Wuhan, China; 4 Department of Pathology, The Center Hospital of Ezhou, Ezhou, China

**Keywords:** Gastric cancer, TPT1-AS1, prognosis, G1/S transition, epithelial–, mesenchymal transition, EMT

## Abstract

Long non-coding RNAs are considered to be critical regulators of tumor progression. Tumor protein translationally controlled 1 antisense RNA 1 (TPT1-AS1) was shown to have an oncogenic role in cervical and ovarian cancer. The clinical significance and biological function of TPT1-AS1 in gastric cancer (GC) are not clear. In this study, we analyzed the expression of TPT1-AS1 in GC tissues and cell lines and performed functional and mechanistic analysis of TPT1-AS1 effects on GC cell proliferation, migration, and invasion. TPT1-AS1 expression was determined in 76 pairs of GC tissues vs. matched adjacent normal tissues and in four GC cell lines (SGC-7901, AGS, BGC-823, and MGC-803) vs. GES-1 cell line by quantitative reverse transcription PCR. SGC-7901 and MGC-803 cells were transfected with small interfering RNA or scrambled negative control, and cell proliferation, colony formation, migration, invasion and cell cycle assays were performed. The expression of proteins involved in cell cycle progression and epithelial–mesenchymal transition was analyzed by Western blot. TPT1-AS1 expression was significantly higher in GC tissues and cell lines compared to controls. The overexpression of TPT1-AS1 was significantly correlated with TNM stage and lymph node metastasis, and it was associated with worse prognosis of GC patients according to the Kaplan–Meier survival analysis and Cox proportional hazard regression analysis. The knockdown of TPT1-AS1 significantly inhibited proliferation, cell cycle G1/S transition, migration, and invasion of SGC-7901 and MGC-803 cells. Moreover, TPT1-AS1 knockdown downregulated the expression of cyclin-dependent kinase (CDK) 4, cyclin D1, and vimentin and upregulated the expression of p21 and E-cadherin. Our findings suggest that TPT1-AS1 may be a promising therapeutic target in GC.

## INTRODUCTION

Gastric cancer (GC) is considered to be one of the most frequently diagnosed malignant tumors and the third leading cause of cancer-associated deaths in the world [1]. According to the latest GLOBOCAN statistics, it was estimated that the incidence rate ranks the fifth with approximately 1.033 million new cases of GC and the mortality rate ranks the second with 783,000 death cases among these new cases in 2018 worldwide [2]. The treatments of GC are challenging owing to the fact that most of the patients present with non-specific symptoms and usually are diagnosed in an advanced stage, causing disappointing clinical outcomes [3,4]. Despite clinical investigators have put forward some risk factors, including dietary habits, drinking and heredity, to be implicated in the initiation and progression of GC, these factors are far from enough for accurate diagnosis and reliable prognosis prediction [5,6]. Therefore, a better understanding of the molecular mechanisms underlying GC pathogenesis is of great importance to develop promising therapeutic strategies and improve the prognosis in GC patients.

Long noncoding RNAs (lncRNAs) are defined as a novel group of transcripts with a length of more than 200 nucleotides [7]. LncRNAs play critical roles in various biological processes, including cellular differentiation, proliferation, cell cycle regulation, migration, invasion, and stem-cell biology [8-10]. In recent years, aberrant expression of lncRNAs has been identified as a crucial biomarker involved in a diverse range of malignancies, including GC. For example, Wang et al. showed that CASC19 was upregulated in advanced GC clinical samples and significantly associated with higher pathologic tumor-node-metastasis (TNM) stage, lymph node metastasis, and poor overall survival (OS) [11]. Amine oxidase copper containing 4 (AOC4P) has been shown to facilitate the proliferation, migration, and invasion of MGC-803 and BGC-823 cells [12]. Conversely, growth arrest associated lncRNA 1 (GASL1) has been reported to be significantly downregulated in GC tissues and to inhibit GC cell proliferation and tumor growth by inactivating the Wnt/β-catenin signaling pathway [13]. Emerging studies show that antisense lncRNAs (lncRNAs-AS) can modulate cancer progression by functioning as positive or negative regulators of coding genes [14-17]. Tumor protein translationally controlled 1 antisense RNA 1 (TPT1-AS1) has been studied by Jiang et al. who showed that TPT1-AS1 as an oncogenic lncRNA promotes cell growth and metastasis in cervical cancer [18], as well as by Wu et al. who further revealed that ectopic TPT1-AS1 expression is strongly associated with unfavorable epithelial ovarian cancer (EOC) clinicopathological features and induces EOC tumor growth and metastasis [19]. Nevertheless, the clinicopathological feature and biological function of TPT1-AS1 in GC remain largely unknown.

In the present study, we determined the expression pattern of TPT1-A1 in GC tissues and cell lines. We further investigated the association between TPT1-AS1 and clinicopathological features of GC, as well as the prognostic role of TPT1-AS1 in GC patients. Moreover, functional experiments were performed to explore the biological function of TPT1-AS1 in GC cell proliferation, migration and invasion, and the underlying regulatory mechanisms, which are expected to provide a theoretical basis for GC treatment.

## MATERIALS AND METHODS

### Patient tissue samples

Paired tumor tissue and corresponding adjacent normal tissues were obtained from 76 patients with GC after surgical resection at The Center Hospital of Ezhou, Ezhou (Hubei, China). Before operation, each patient signed the written consent statement and did not receive preoperative chemo or radiotherapy. All fresh tissue samples were frozen and stored at -80°C until further analysis. The main clinicopathological characteristics, including gender, tumor size and TNM stage, were recorded (Table 1) and the interval between the date of surgery (18 April 2014) and final clinical follow-up was calculated as OS. This study was approved by the Ethics Committee of The Center Hospital of Ezhou in accordance with the Helsinki Declaration.

**TABLE 1:**
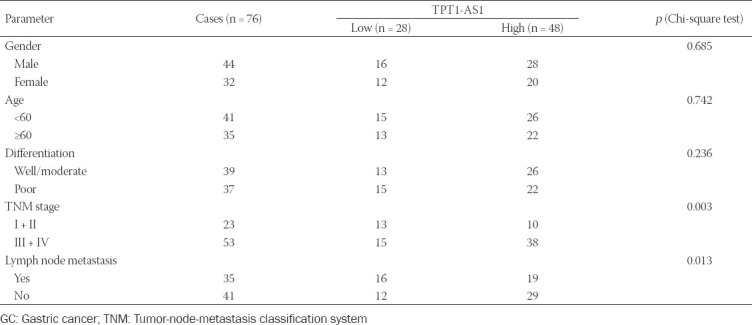
Association of TPT1-AS1 expression with clinicopathological features in GC (n = 76)

### Cell culture and transfection

Four human GC cell lines (SGC-7901, AGS, BGC-823, and MGC-803) and a normal gastric mucosal epithelial cell line (GES-1) were purchased from Cell Bank of Chinese Academy of Sciences (Shanghai, China) and cultivated in RPMI 1640 medium (HyClone, Logan, UT, USA) supplemented with 10% fetal bovine serum (FBS) at 37°C in a humidified atmosphere containing 5% CO_2_. The small interfering RNA targeting TPT1-AS1 (si-TPT1-AS1) and a scrambled negative control (si-NC) were synthesized by RiboBio (Guangzhou, China), which were transfected into SGC-7901 and MGC-803 cells at a density of 3.0 × 10^5^ cells per well in 6-well plates by the Lipofectamine 3000 Reagent (Invitrogen, Carlsbad, CA, USA).

### Quantitative reverse transcription polymerase chain reaction (qRT-PCR)

Total RNA was isolated from tissues or cell lines using TRIzol reagent (Invitrogen) and cDNA was synthesized by a PrimeScript™ RT Master Mix Kit (TaKaRa BIO, Japan). In accordance with the instructions of SYBR-Green PCR Master Mix Kit (Takara, China), the expression of TPT1-AS1 was determined on an ABI PRISM 7700 Sequence Detection System (Applied Biosystems, Foster City, CA, USA) and calculated by the 2^−ΔΔCt^ method with glyceraldehyde 3-phosphate dehydrogenase (GAPDH) as an internal control. The primer sequences used in PCR were as follows: TPT1-AS1 forward: 5'-AGGAGGCTATCCTTGCCCATC-3' and reverse: 5'-AATTGGAGGCCAGTGCTCTGAA-3'; GAPDH forward: 5'-TGGTATCGTGGAAGGACTCAT-3' and reverse: 5'-GTGGGTGTCGCTGTTGAAGTC-3'.

### CCK-8 assay

To investigate the effect of TPT1-AS1 on GC cell proliferation, SGC-7901 and MGC-803 cells were seeded into 96-well plates at a density of 3000 cells/well. After culturing for 24, 48 and 72 h, 10 μl of CCK-8 solution (Dojindo, Japan) was added into each well, and cells were incubated for another 2 h. Subsequently, the optical density (OD) at a wavelength of 450 nm was measured using a microplate reader (BioTek Instruments, VT, USA).

### Colony formation assay

SGC-7901 and MGC-803 cells at a density of 500 cells per well were seeded into 6-well plates and cultured for consecutive 2 weeks. The naturally formed colonies were fixed with 4% paraformaldehyde (Beyotime, Shanghai, China) for 30 min and stained with 0.1% crystal violet (Sigma-Aldrich, St. Louis, MO, USA) for 15 min. The colonies (50 cells per colony) were observed and quantified under a microscope.

### Flow cytometry analysis

Cell cycle distribution and apoptosis were analyzed by flow cytometry. In brief, SGC-7901 and MGC-803 cells were digested with trypsin, resuspended in phosphate-buffered saline (PBS) and collected in centrifuge tubes, and treated with 70% ice-cold ethanol overnight at 4°C. For cell cycle assay, fixed cells were washed twice with PBS, stained with propidium iodide (PI, Sigma-Aldrich) for 30 min at room temperature and analyzed by a FACSCalibur flow cytometer (BD Biosciences, Franklin Lakes, USA).

### Transwell assays

Transwell migration and invasion assay were performed in SGC-7901 and MGC-803 cells using 24-well transwell chamber inserts (Corning Inc., Corning, NY, USA). Briefly, cells were resuspended in serum-free medium and seeded in the upper chambers with or without Matrigel (BD Biosciences, San Jose, CA, USA). The lower chamber was filled with medium containing 10% FBS as a chemoattractant. After 24 h incubation, the cells that migrated or invaded into the lower chamber were fixed with 70% ethanol for 30 min and stained with 0.1% crystal violet for 10 min, followed by quantification in randomly selected five fields under a microscope.

### Western blot analysis

SGC-7901 and MGC-803 cells were rinsed with ice-cold PBS and lysed in RIPA buffer (Beyotime Biotechnology, Shanghai, China). The extracted protein samples were analyzed using a BCA assay (Beyotime Biotechnology), separated by 10% sodium dodecyl sulfate-polyacrylamide gels (SDS-PAGE) and then transferred onto polyvinylidene difluoride (PVDF) membranes (Millipore, MA, USA). The membranes were blocked in 5% non-fat milk supplemented with Tris-buffered saline containing 0.1% Tween-20 (TBST) and incubated with primary antibodies against cyclin-dependent kinase (CDK) 4, cyclin D1, p21, E-cadherin, N-cadherin, and GAPDH at 4°C overnight. After rinsing 3 times with TBST, the membranes were incubated with horseradish peroxidase-linked secondary antibodies at room temperature for 2 h. Protein bands were visualized with an enhanced chemiluminescence kit (Millipore).

### Statistical analysis

Statistical analysis was conducted using IBM SPSS Statistics for Windows, Version 19.0. (IBM Corp., Armonk, NY, USA). The associations between TPT1-AS1 and clinicopathological features were analyzed by Chi-square test. The effects of TPT1-AS1 on OS were evaluated by Kaplan–Meier method and the log-rank test. The Cox regression analysis was performed to assess the possible factors that can predict the prognosis of GC patients. The data from the *in vitro* experiments were expressed as mean ± standard deviation. Student’s *t*-test was applied to compare the differences between two groups. Differences among multiple groups were assessed by one-way analysis of variance followed by Tukey’s *post hoc* test. The level of statistical significance was set at *p* < 0.05.

## RESULTS

### Significantly elevated TPT1-AS1 expression predicted poor prognosis of GC patients

**FIGURE 1:**
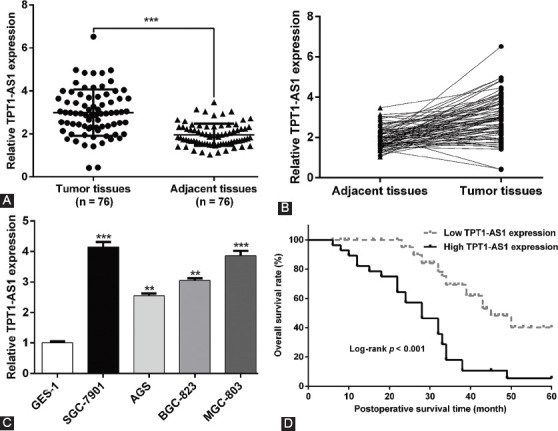
The expression of TPT1-AS1 in GC and its correlation with patient survival. (A and B) The TPT1-AS1 expression was analyzed in 76 pairs of GC tissue samples and matched adjacent normal tissues using quantitative reverse transcription PCR (qRT-PCR). ***p < 0.001 compared with adjacent tissues; (C) TPT1-AS1 expression was measured in four GC cell lines (SGC-7901, AGS, BGC-823, and MGC-803) and normal gastric mucosal epithelial cell line GES-1 by qRT-PCR. **p < 0.01, ***p < 0.001, compared with GES-1; (D) Kaplan–Meier survival curves were plotted to assess the association between TPT1-AS1 expression and 5-year survival rate in GC patients, which indicated that GC patients with low TPT1-AS1 expression have a better overall survival rate. GC: Gastric cancer; TPT1-AS1: Tumor protein translationally controlled 1 antisense RNA 1.

We first analyzed the expression profile of TPT1-AS1 using qRT-PCR in 76 paired GC tissues and adjacent normal tissues. As depicted in Figure 1A and B, TPT1-AS1 expression was significantly increased in GC tissues compared with adjacent normal tissues. Consistently, the expression of TPT1-AS1 was markedly upregulated in four GC cell lines (SGC-7901, AGS, BGC-823, and MGC-803) compared with normal GES-1 (Figure 1C). Besides, patients were divided into high TPT1-AS1 expression group (n = 48) and low TPT1-AS1 expression group (n = 28) according to the median expression value. The association between TPT1-AS1 expression and clinicopathological features was analyzed by Chi-square test. As described in Table 1, we observed that TPT1-AS1 expression level was significantly associated with TNM stage (*p* = 0.003) and lymph node metastasis (*p* = 0.013). The Kaplan–Meier survival curves and log-rank test demonstrated that GC patients with high TPT1-AS1 expression had a relative poor OS rate than that with low TPT1-AS1 expression (Figure 1D). Moreover, the Cox proportional hazard regression analysis revealed TPT1-AS1 expression (HR = 0.853, 95% CI: 0.509–0.997, *p* = 0.015) was the potential dependent risk factor affecting the OS of GC patients (Table 2). These data strongly suggested that high TPT1-AS1 expression was correlated with poor clinical prognosis in GC patients.

**TABLE 2:**
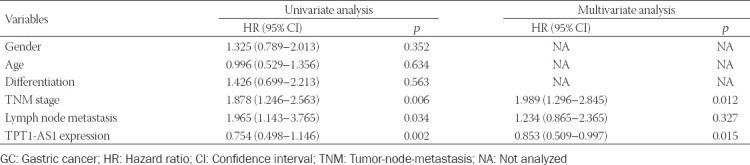
Cox regression analysis of prognostic factors affecting overall survival in GC patients

### Downregulation of TPT1-AS1 inhibited GC cell proliferation and induced cell cycle G0/G1 phase arrest

**FIGURE 2:**
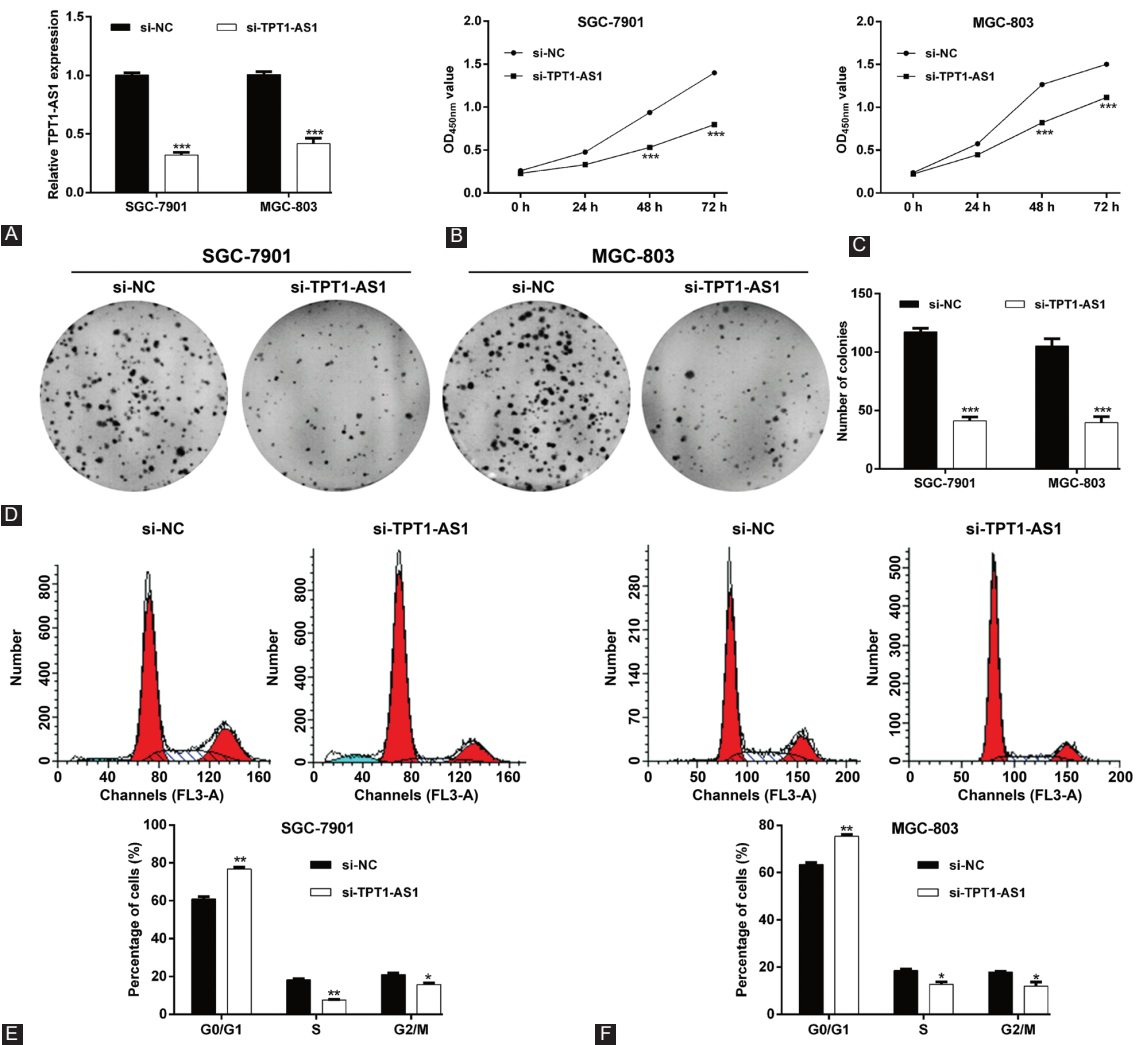
Effects of TPT1-AS1 knockdown on GC cell proliferation and cell cycle distribution. SGC-7901 and MGC-803 cells were transfected with si-TPT1-AS1 or si-NC for 48 h. (A) TPT1-AS1 expression was determined in transfected SGC-7901 and MGC-803 cells using quantitative reverse transcription PCR. CCK-8 assay was utilized to analyze cell proliferation ability of transfected SGC-7901 (B) and MGC-803 (C) cells. (D) Cell colony formation was assessed in transfected SGC-7901 and MGC-803 cells. Flow cytometry assay was carried out to analyze the distribution of cells at G0/G1, S, and G2/M phases in transfected SGC-7901 (E) and MGC-803 (F) cells. *p < 0.05, **p < 0.01, ***p < 0.001, compared with si-NC. GC: Gastric cancer; TPT1-AS1: Tumor protein translationally controlled 1 antisense RNA 1; si-TPT1-AS1: Small interfering RNA targeting TPT1-AS1; si-NC: Scrambled negative control.

Compared with AGS and BGC-823 cells, SGC-7901 and MGC-803 cells showed relatively higher TPT1-AS1 expression and were thus chosen as the model to explore the biological function of TPT1-AS1 in GC cells. The expression of TPT1-AS1 was significantly reduced in SGC-7901 and MGC-803 cells after transfection with si-TPT1-AS1 (Figure 2A). The CCK-8 assay showed that the downregulation of TPT1-AS1 by si-TPT1-AS1 transfection greatly suppressed the proliferative ability of SGC-7901 (Figure 2B) and MGC-803 (Figure 2C) cells. In line with this, we observed si-TPT1-AS1 transfection significantly decreased the number of colonies compared with si-NC transfection in both SGC-7901 and MGC-803 cells (Figure 2D). Moreover, we investigated whether si-TPT1-AS1-impeded GC cell proliferation was associated with cell cycle regulation using flow cytometry. As shown in Figure 2E, the distribution of si-TPT1-AS1-transfected SGC-7901 cells was noticeably elevated in G0/G1 phase (76.8% ± 0.9% vs. 60.8% ± 1.3%), and accordingly reduced in S phase (7.5% ± 0.4% vs. 18.2% ± 0.6%) and G2/M phase (15.7% ± 0.8% vs. 21.0% ± 0.9%) in comparison with si-NC-transfected SGC-7901 cells. Similarly, the downregulation of TPT1-AS1 induced cell cycle G0/G1 phase arrest in MGC-803 cells (Figure 2F).

### Downregulation of TPT1-AS1 attenuated GC cell migration and invasion

**FIGURE 3:**
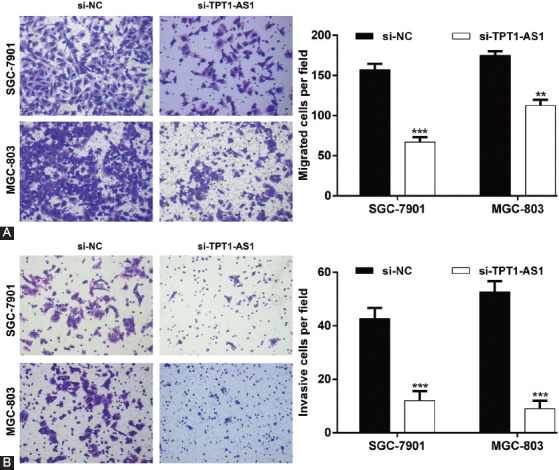
Effects of TPT1-AS1 knockdown on GC cell migration and invasion. The migration (A) and invasion (B) of SGC-7901 and MGC-803 cells after transfection with si-TPT1-AS1 or si-NC were assessed by Transwell assays. The number of migratory and invasive cells was quantified as cell numbers (×200 magnification). **p < 0.01, ***p < 0.001, compared with si-NC. GC: Gastric cancer; TPT1-AS1: Tumor protein translationally controlled 1 antisense RNA 1; si-TPT1-AS1: Small interfering RNA targeting TPT1-AS1; si-NC: Scrambled negative control.

Next, a Transwell assay was performed to examine the effect of TPT1-AS1 on migration and invasion in GC cells. As depicted in Figure 3A, the downregulation of TPT1-AS1 remarkably decreased the number of migrated cells from 157.0 ± 7.5 to 67.0 ± 6.2 in SGC-7901 cells and from 175.0 ± 5.0 to 112.7 ± 7.0 in MGC-803 cells. In addition, the invasion ability of GC cell was significantly suppressed, as demonstrated by a decreased number of invasive cells in si-TPT1-AS1 group compared with si-NC group in both SGC-7901 (12.0 ± 3.6 vs. 42.7 ± 4.0) and MGC-803 (9.0 ± 3.0 vs. 52.7 ± 4.0) cells (Figure 3B). These data indicated that the cell migration and invasion ability was weakened in GC cells following TPT1-AS1 downregulation.

### Downregulation of TPT1-AS1 regulated the key regulators of cell cycle G1/S transition and epithelial–mesenchymal transition (EMT)

**FIGURE 4:**
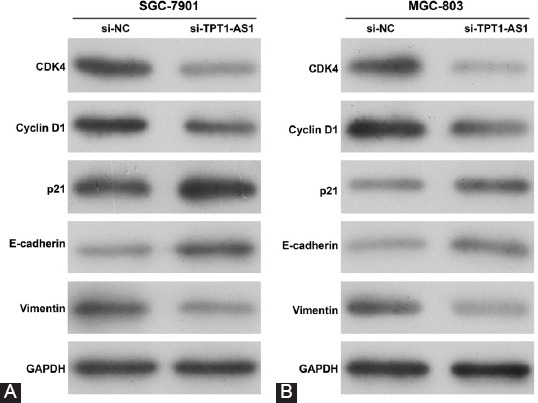
Effects of TPT1-AS1 knockdown on the key regulators in cell cycle G1/S transition and epithelial–mesenchymal transition (EMT). SGC-7901 and MGC-803 cells were transfected with si-TPT1-AS1 or si-NC for 48 h. The protein expression of CDK4, cyclin D1, p21, E-cadherin, and N-cadherin was measured by Western blot in transfected SGC-7901 (A) and MGC-803 (B) cells. GC: Gastric cancer; TPT1-AS1: Tumor protein translationally controlled 1 antisense RNA 1; si-TPT1-AS1: Small interfering RNA targeting TPT1-AS1; si-NC: Scrambled negative control; CDK: Cyclin-dependent kinase; GAPDH: Glyceraldehyde 3-phosphate dehydrogenase.

To further confirm the suppressive effects of si-TPT1-AS1 on cell cycle progression, migration and invasion ability in GC cells, we analyzed the key regulators associated with cell cycle G1/S transition and EMT. The results showed CDK4 and Cyclin D1, associated with G1/S transition, were both downregulated, while p21 as a CDK inhibitor was upregulated after TPT1-AS1 knockdown in SGC-7901 cells (Figure 4A). We also found TPT1-AS1 knockdown obviously increased the expression of the epithelial marker E-cadherin and reduced the expression of the mesenchymal marker vimentin in SGC-7901 cells (Figure 4A). The same protein expression levels of CDK4, cyclin D1, p21, E-cadherin, and vimentin were consistently validated in MGC-803 cells after TPT1-AS1 knockdown (Figure 4B). These findings showed that TPT1-AS1 acts as an important regulator in the promotion of cell cycle progression and EMT-mediated migration and invasion in GC cells.

## DISCUSSION

An increasing amount of evidence has showed that lncRNAs are crucial regulators of the biological processes involved in the occurrence and development of GC [20,21]. Here, we first found TPT1-AS1, an antisense lncRNA, was significantly upregulated in GC tissues and cell lines, which was significantly associated with TNM stage, lymph node metastasis, and worse prognosis. These findings indicated that TPT1-AS1 may participate as an oncogene in the pathogenesis of GC. Consistently, TPT1-AS1 was remarkably elevated in cervical cancer [18] and ovarian cancer tissues [19]. Moreover, elevated TPT1-AS1 expression predicted worse prognosis in ovarian cancer patients [19]. On the contrary, TPT1-AS1 was found to be decreased and identified as a protective lncRNA in glioblastoma [22]. These previous studies suggest that TPT1-AS1 holds different functions in different tumors.

We next performed loss-of-function assays to elucidate the biological function of TPT1-AS1 by selecting SGC-7901 and MGC-803 cells, exhibiting relatively higher TPT1-AS1 expression, as a model. The results showed that the knockdown of TPT1-AS1 suppressed cell proliferation, migration and invasion, and induced cell cycle arrest in GC cells. Antisense lncRNAs comprise transcripts with sequence complementarity to other RNA, which have been widely proven to be overexpressed in various tumors and cell lines [23]. For instance, BNC2-AS1 knockdown markedly suppressed GC cell proliferation and migration [24]. Elevated expression of NNT-AS1 promoted GC cell proliferation, cell cycle progression, and invasion [25]. Another study also demonstrated that TP73-AS1 accelerates cell growth and metastasis in GC [26]. Interestingly, TPT1-AS1 was proved to promote ovarian cancer tumor growth and metastasis through the downstream PI3K/AKT signaling pathway [19].

Furthermore, we attempted to explore the molecular mechanisms underlying TPT1-AS1 regulation of cell cycle G1/S transition, migration, and invasion. Our results showed that CDK4 and Cyclin D1 were downregulated, while p21 was upregulated in the cell cycle G0/G1 phase arrest induced by TPT1-AS1 knockdown in GC cells. CDK4/CDK6-cyclin D complexes and CDK inhibitors are required for cell cycle transition from the G0/G1 to S phases [27,28]. As one of CDK inhibitors, p21 could inhibit the activities of CDKs and cyclins in the G1/S phase by binding to their subunits through its characteristic motifs within amino-terminal moieties [29]. Thus, the suppressive effects of TPT1-AS1 knockdown on GC cell proliferation might be partially ascribed to si-TPT1-AS1-induced cell cycle G0/G1 phase arrest. Furthermore, we observed increased epithelial marker E-cadherin and reduced mesenchymal marker vimentin expression in GC cells following TPT1-AS1 knockdown. EMT plays an important role in tumor invasion and metastasis, which is characterized as loss of epithelial phenotype and the acquisition of mesenchymal properties, causing enhanced migratory and invasion ability [30-32]. Here, TPT1-AS1 knockdown caused upregulation of E-cadherin expression and downregulation of vimentin expression, providing an evidence of EMT inhibition. Therefore, targeting the EMT process is presently considered to be another molecular mechanism underlying TPT1-AS1 knockdown, decreasing cell migration and invasion.

## CONCLUSION

Collectively, our study revealed that TPT1-AS1 overexpression serves as a prognostic factor of adverse clinical features and worse prognosis in GC patients. We also revealed the involvement of TPT1-AS1 in promoting cell proliferation, migration, and invasion of GC cells, possibly by regulating the cell cycle G1/S transition and EMT. These findings provide a new understanding of TPT1-AS1 role in GC and may help develop promising targets for the molecular treatment of GC.
